# Impact of Meteorological Factors and Southern Oscillation Index on Scrub Typhus Incidence in Guangzhou, Southern China, 2006–2018

**DOI:** 10.3389/fmed.2021.667549

**Published:** 2021-07-28

**Authors:** Jianyun Lu, Yanhui Liu, Xiaowei Ma, Meixia Li, Zhicong Yang

**Affiliations:** ^1^Department of Infectious Disease Control and Prevention, Guangzhou Center for Disease Control and Prevention, Guangzhou, China; ^2^Department of Public Health Emergency Preparedness and Response, Guangzhou Center for Disease Control and Prevention, Guangzhou, China; ^3^Guangzhou Center for Disease Control and Prevention, Guangzhou, China

**Keywords:** distributed lag non-linear models, meteorological factors, southern oscillation index, scrub typhus, early warning

## Abstract

**Background:** Scrub typhus was epidemic in the western Pacific Ocean area and East Asia, scrub typhus epidemic in densely populated areas in southern China. To better understand the association between meteorological variables, Southern Oscillation Index (SOI), and scrub typhus incidence in Guangzhou was benefit to the control and prevention.

**Methodology/Principal Findings:** We collected weekly data for scrub typhus cases and meteorological variables in Guangzhou, and Southern Oscillation Index from 2006 to 2018, and used the distributed lag non-linear models to evaluate the relationships between meteorological variables, SOI and scrub typhus. The median value of each variable was set as the reference. The high-risk occupations were farmer (51.10%), house worker (17.51%), and retiree (6.29%). The non-linear relationships were observed with different lag weeks. For example, when the mean temperature was 27.7°C with1-week lag, the relative risk (RR) was highest as 1.08 (95% CI: 1.01–1.17). The risk was the highest when the relative humidity was 92.0% with 9-week lag, with the RR of 1.10 (95% CI: 1.02–1.19). For aggregate rainfall, the highest RR was 1.06 (95% CI: 1.03–1.11), when it was 83.0 mm with 4-week lag. When the SOI was 19 with 11-week lag, the highest RR was 1.06 (95% CI: 1.01–1.12). Most of the extreme effects of SOI and meteorological factors on scrub typical cases were statistically significant.

**Conclusion/Significance:** The high-risk occupations of scrub typhus in Guangzhou were farmer, house worker, and retiree. Meteorological factors and SOI played an important role in scrub typhus occurrence in Guangzhou. Non-linear relationships were observed in almost all the variables in our study. Approximately, mean temperature, and relative humidity positively correlated to the incidence of scrub typhus, on the contrary to atmospheric pressure and weekly temperature range (WTR). Aggregate rainfall and wind velocity showed an inverse-U curve, whereas the SOI appeared the bimodal distribution. These findings can be helpful to facilitate the development of the early warning system to prevent the scrub typhus.

## Introduction

Scrub typhus (tsutsugamushi disease), a rickettsiosis caused by *Orientia tsutsugamushi*, is epidemic in the western Pacific Ocean area and East Asia (1, 2). Recently, scrub typhus cases showed a rising trend in Asia, case-fatality rates from areas with reduced drug sensitivity were reported in South India and northern Thailand at 12.2 and 13.6%, respectively (3), and it has also become an important health issue in China (4). At present, scrub typhus epidemic in densely populated areas in southern China and millions of people is at risk of the infection of scrub typhus (5). A rapid increase of scrub typhus was reported in Guangzhou in 2012, which was a nearly four-fold increase from 2006 (5). A rising trend was observed in recent years, which caused a huge disease burden in Guangzhou city. Several reasons might contribute to the increase of scrub typhus cases, such as variation of genotypes of *O. tsutsugamushi* (6), increased risk of exposure to vegetation with more and more parks in the city construction (7), improvement of the surveillance system, and climate change. A previous study showed that climate factors played an important role in the spread of vector-borne diseases (8).

In common, human is transmitted by the bite of infected larval trombiculid mites, with the incubation period about 4 to 21 days. Fever, eschars or ulcers, skin rash, and lymphadenopathy are typical clinical symptoms (9). Orientia-infected mites inhabit in various environments, including grassland, field, forest, and scrub. Transmission of scrub typhus rests with the seasonal activities of both chiggers and humans (10). Hence, scrub typhus mainly occurred in farmers and urban populations who have an exposure in parks and the countryside (11).

For the past few years, the effects of meteorological variables have been broadly studied as early-warning signals of underlying epidemics on some rodent-borne infectious diseases, such as, hemorrhagic fever with renal syndrome (12), lime-disease and tick-borne encephalitis (13). Previous studies showed that the transmission of scrub typhus influenced by climate. For instance, high rainfall and temperature could increase the chigger abundance, which increased the spread of scrub typhus (5, 7). El Niño/Southern Oscillation (ENSO) is a systematic pattern of global climate variability (14). The Southern Oscillation Index (SOI), an indicator of ENSO activity, is defined as the normalized atmospheric pressure difference between Darwin, Australia and Tahiti, South Pacific (15). The SOI has been reported to be associated with infectious diseases, including hemorrhagic fever with renal syndrome (16), and dengue fever (17). However, limited researches reported the weekly associations between climatic variables, SOI, and scrub typhus cases. Moreover, the quantitative and lagged relationships mentioned above remain to be determined. Hence, it's urgent to detect these associations to build up forecasting and early warning for scrub typhus, especially in Guangzhou, southern China, with the nationwide highest scrub typhus incidence (4, 5).

In our research, we used the distributed lag non-linear models (DLNMs), which can flexibly describe relationships and explore underlying lagged and non-linear effects (18), to detect the association between SOI, meteorological variables, and scrub typhus cases. Our findings can not only offer in-depth insights into the future effect of climate change on the transmission of scrub typhus in southern China, but also in subtropical zone.

## Materials and Methods

### Study Region

Guangzhou is the third largest city in China, which has a registered inhabitant over 8.97 million. Guangzhou, located in southern China, has a humid subtropical climate with hot and wet summers, and cool, dry winters, which locates at 112°57'E to 114°3'E and 22°26' N to 23°56'N.

### Data of Scrub Typhus Cases

During the period from 2006 to 2018, data of scrub typhus cases in Guangzhou were obtained from the National Notifiable Disease Report System, which covers all community health centers and hospitals in Guangzhou. The clinical diagnosis cases should meet the criteria as follows. (1) Fever with lymphadenopathy or skin rash, (2) typical eschars or ulcers, and (3) A history of field exposure 1-3 weeks before the onset of symptoms. The laboratory confirmed cases must meet the criteria mentioned above and fulfill one of the following criteria: four-fold or more increase in serum IgG antibody titers between acute and convalescent sera-detected by indirect immune fluorescence antibody assay (IFA); The *O. tsutsugamushi* was detected in clinical specimens using polymerase chain reaction (PCR); the isolation of *O. tsutsugamushi* (19). The cases in our study contained clinical cases and laboratory confirmed cases. Scrub typhus was not the national notifiable disease; however, it required compulsory notification for doctors in Guangzhou by the local laws.

### Meteorological Data

We collected the weekly meteorological data, such as mean temperature (°C), aggregate rainfall (mm), weekly temperature range (°C) (WTR: it is defined as the gap between the highest and the lowest temperature in a week), wind velocity (m/s), relative humidity (%), atmospheric pressure (hPa), and sunshine hours (hours), were collected from the Guangzhou Meteorological Bureau (GZMB), which measure the meteorological data at a fixed-site station located in 186 surveillance stations in different regions of all the 11 districts of Guangzhou city. SOI data were publicly accessible from the Australian Bureau of Meteorology (http://www.longpaddock.qld.gov.au/).

### Statistical Analysis

Based on the descriptive analysis of scrub typhus cases, SOI and meteorological variables in Guangzhou, we deduced that their correlations were non-linear, which the DLNMs were suitable to detect. We used the variance inflation factor (VIF) to evaluate the co-linearity. If the VIF exceeds 5, it would be manifest multicollinearity (20). All the predictor variables studied in our study were tested for each model.

The quasi-Poisson function was incorporated with DLNMs, allowing over-dispersion in the scrub typhus cases. The model structure is as below:


$$\begin{array}{l} \text{Log}\left[\text{E}\left({\text{Y}}_{\text{t}}\right)\right]=\alpha +\sum NS_{\text{i}}\left({\text{X}}_{\text{i}},{\text{df}}_{\text{i}}\right)+\delta {\text{Holiday}}_{\text{t}}+\text{NS}\left(\text{Time},14\right)+\Phi_{\text{t}} \end{array}$$

Yt represents the scrub typhus cases in week t; α and δ are both the intercept; NS represents a natural cubic spline; Holiday is a binary variable, if week t contains more than 7 days of public or summer/winter vacation, its value is “1”; and Time symbolizes long-term trends and seasonality; df means the degree of freedom; X represents meteorological factors and SOI. The incubation of the scrub was from 4 days to 20 days. Φ_t_ refers to the autoregressive terms of weekly flu counts on the logarithmic scale at lag 1 week to control for the autocorrelations occurring in cases of infectious disease (21). We alternated the lag week from 1 to 3 weeks, and finally selected the lag 1 week due to the The Akaike information criterion (AIC).

The AIC was applied to select the df per week for controlling long-term trends and seasonality, and the df for the meteorological variables and the SOI that yielded the best-fitting model. We selected NS (df = 3) for the meteorological factors and the SOI. Different time lags of weeks were applied to evaluate the effects of meteorological variables on scrub typhus according to AIC. The finally model structure for each variable was shown in the Supplementary Table 1.

For all meteorological variables, as the reference of the median value of each variable, we define the extremely high effect as the value in the 97.5% range and extremely low effect as the value in the 2.5% range. We then evaluated these extreme effects on the scrub typhus cases.

The level of significant difference was set as two-sided *P* < 0.05. R software version 3.4.5 was utilized to analyze the data and create the DLNMs by the “dlnm” package.

We performed sensitivity analyses by altering the df (5–9) per year for time, and the df (3–5) for meteorological variables.

## Results

**Figure 1:**
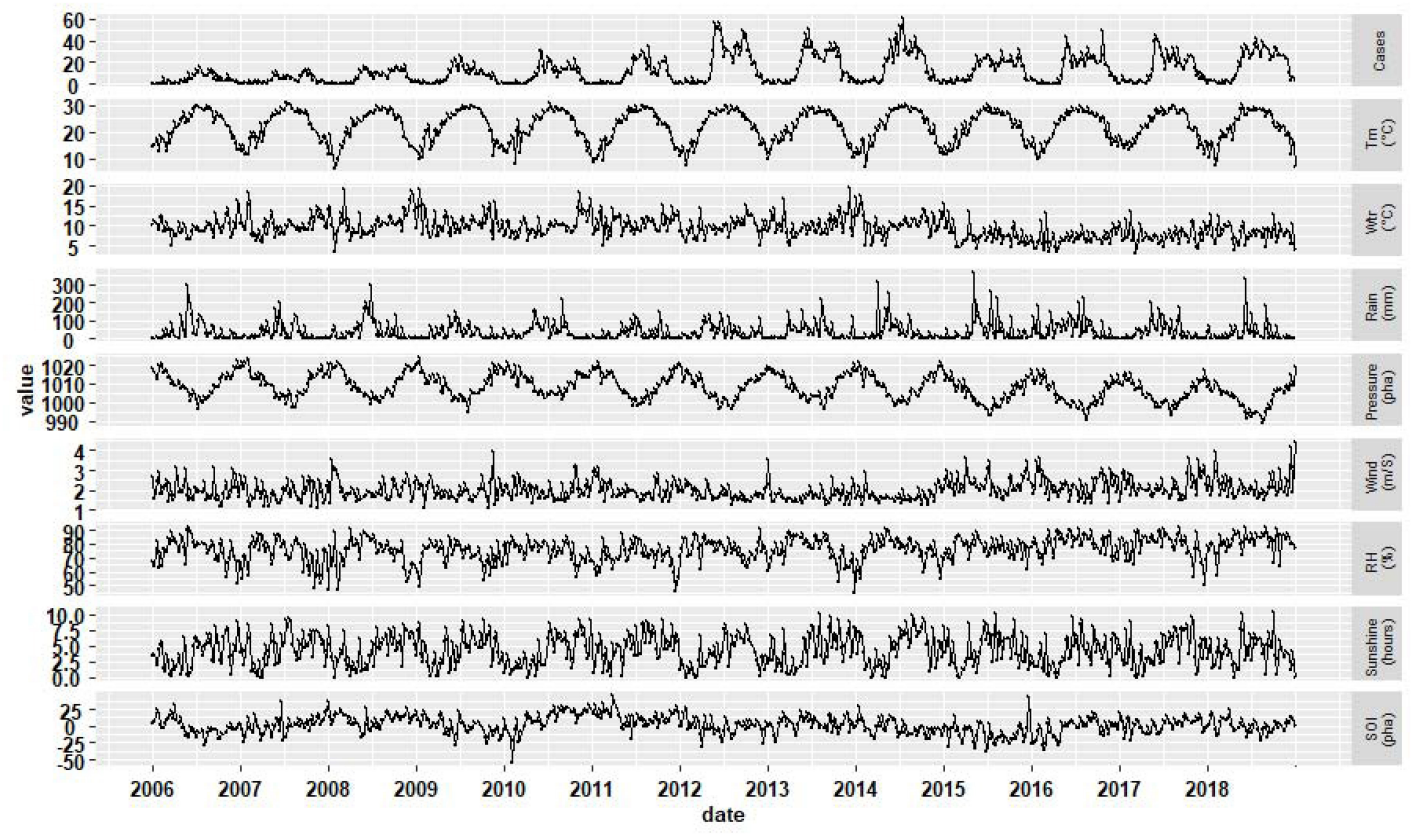
The distribution of weekly scrub typhus cases, meteorological variables and SOI in Guangzhou, Southern China, 2006–2018.

From January 1, 2006 to December 31, 2018, a total of 8,345 scrub typhus cases were reported in Guangzhou. 48.54% (4,051) were male cases and 51.46% (4,294) were female cases, with the male-to-female ratio of 0.94. Most of the cases were clinical diagnosis cases, accounting for 89.87% (7,500). During the study period, the numbers of scrub typhus showed a rising trend, with the top three in 2012 (1,026), 2018 (965), 2014 (936). From 2006 to 2011, cases increased slowly, then the cases had a sharp rise in 2012 and fluctuated, after 2014, the incidence decreased slightly, however it increased again in 2018. The proportion of cases was 5.97% (under 18 years old), 2.70% (from 18 to 24 years old), 56.88% (25 to 60 years old), and 34.45% (over 60 years old), respectively. The high-risk occupations were farmer (51.10%), house worker (17.51%), and retiree (6.29%). During 2006–2018, 84.76% (7,073) cases occurred from May to October, with the peak at June. (Figure 1) during the study period, Table 1 showed the weekly distribution of meteorological variables, SOI and scrub typhus cases.

**Table 1:** Weekly meteorological factors, SOI and scrub typhus cases in Guangzhou, 2006–2018.

**Variables**	**Mean**	**Std. deviation**	**Percentile**			**Min**.	**Max**.
			**25**	**50**	**75**		
Cases	12	12	2	6	19	0	59
Mean temperature	22.21	5.92	17.39	23.45	27.52	6.15	31.13
Weekly temperature range	9.47	2.75	7.64	9.21	10.98	2.97	19.67
Aggregate rainfall	38.87	53.10	1.20	18.50	56.20	0.00	368.30
Atmospheric pressure	1008.18	6.90	1003.03	1008.07	1013.63	988.81	1024.56
Wind velocity	2.02	0.51	1.65	1.94	2.30	1.11	4.45
Relative humidity	76.37	8.64	71.32	77.57	82.46	44.79	92.93
Sunshine hours	4.40	2.54	2.33	4.31	6.38	0	10.57
SOI	1.67	13.49	−6.01	1.85	10.65	−54.04	44.99

**Figure 2:**
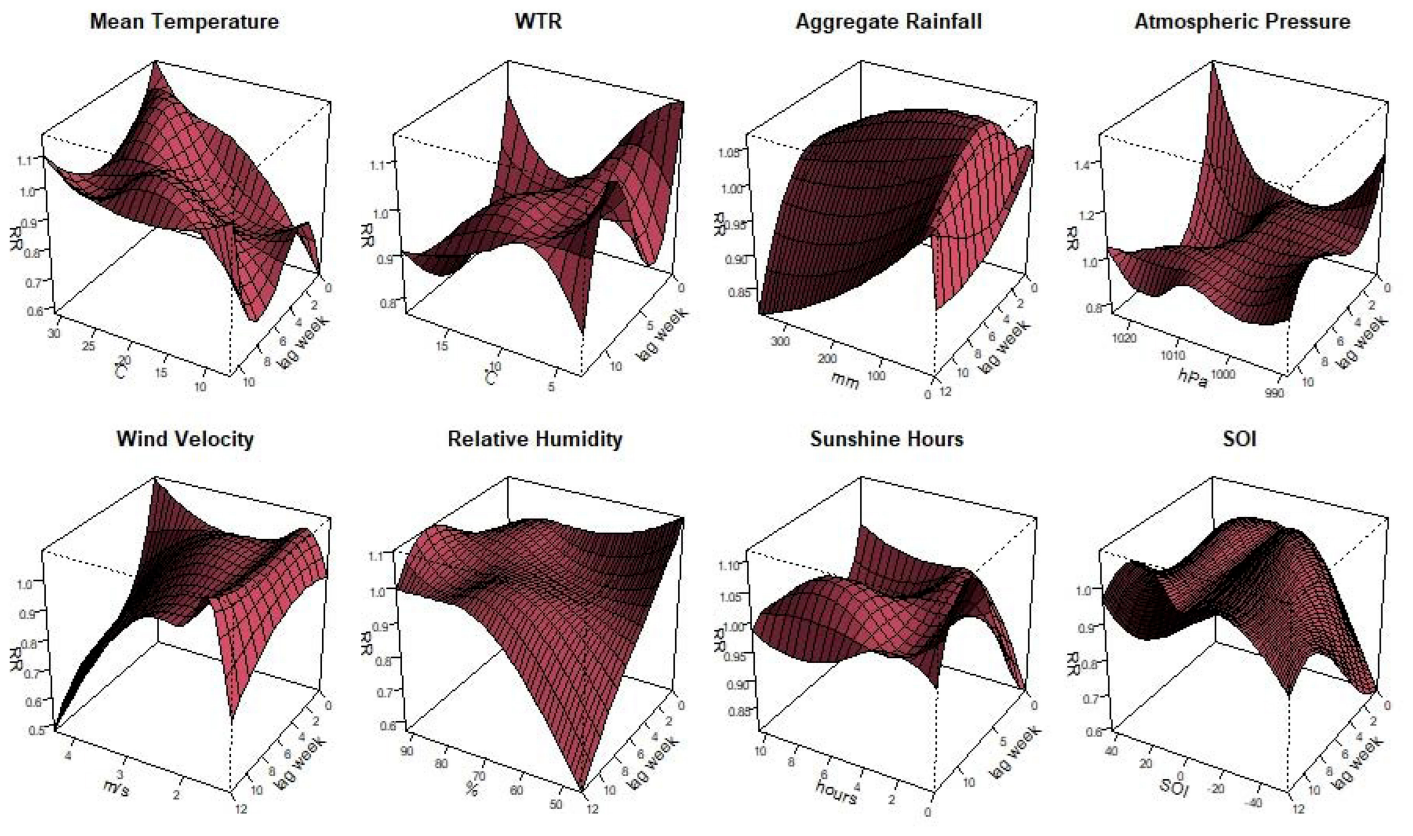
Plots of the relative risk of meteorological factors and the SOI on scrub typhus cases, including mean temperature, diurnal temperature range, aggregate rainfall, air pressure, wind velocity, relative humidity, sunshine hours, and Southern Oscillation Index.

The Figure 2 illustrated the non-linear relationship between the meteorological variables, SOI and scrub typhus cases in Guangzhou with different lag weeks. Different characteristics were observed in the different variables. When the mean temperature was 27.7°C with1-week lag, the relative risk (RR) was highest as 1.08 (95% CI: 1.01–1.17). The risk was the highest when the relative humidity was 92.0% with 9-week lag, with the RR of 1.10 (95% CI: 1.02–1.19). When the WTR was 6.0°C with 0-week lag, the RR reached the high value as 1.10 (95% CI: 1.18–1.20). When the wind speed was 1.6 m/s with 2-week lag, the highest RR was 1.04 (95% CI: 1.00–1.08). For aggregate rainfall, the highest RR was 1.06 (95% CI: 1.03–1.11), when it was 83.0 mm with 4-week lag. As the atmospheric pressure was 989.0 hPa with 0-week lag, the RR reached the peak of 1.29 (95% CI: 1.04–1.61). For the duration of sunshine, the RR was 1.21 (95% CI: 1.05–1.18) when the sunshine was 0 h with 10-week lag. Regarding the SOI, the highest RR was 1.06 (95% CI: 1.01–1.12). When the SOI was 18.8 with 11-week lag.

**Figure 3:**
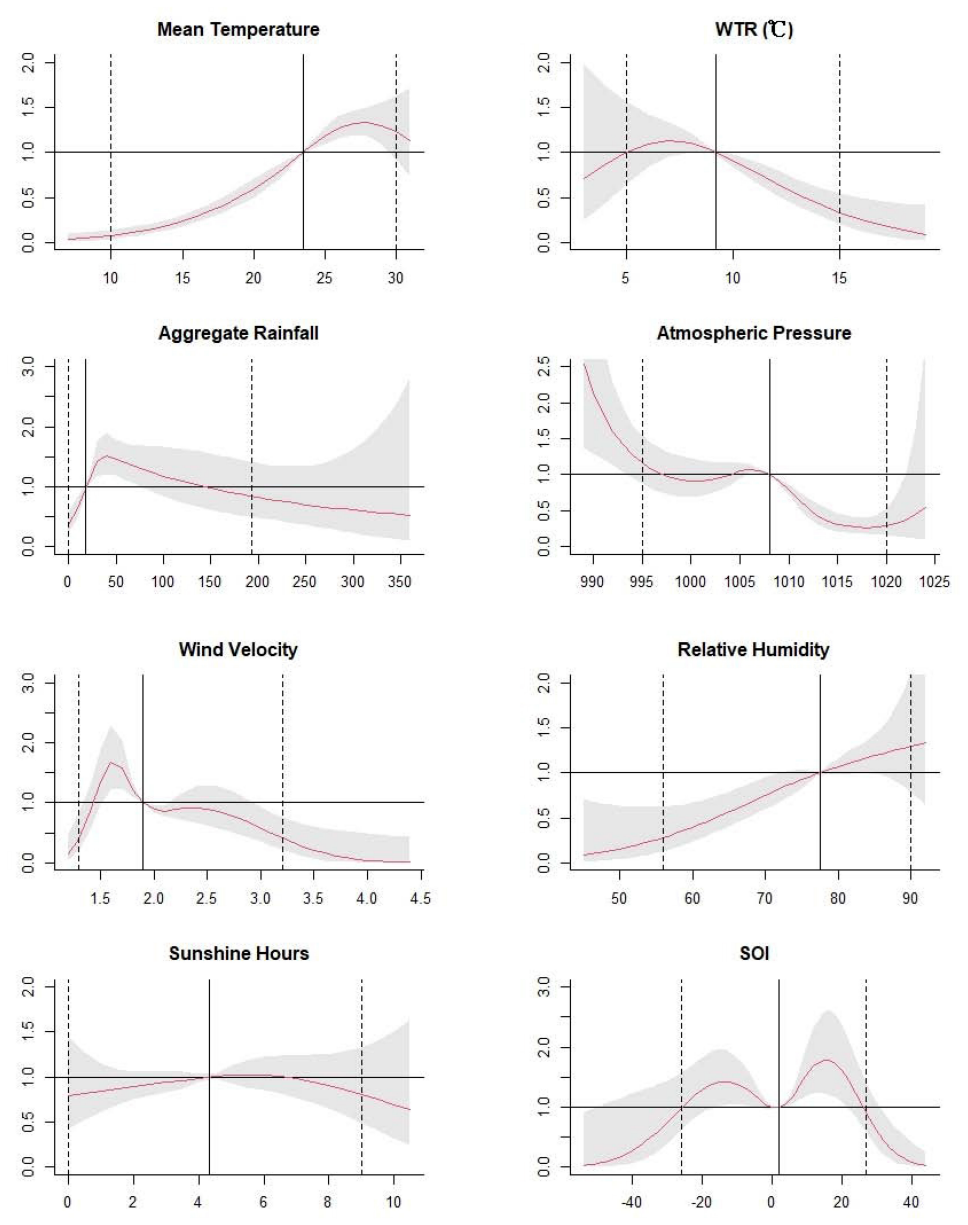
The estimated overall effects of mean temperature, weekly temperature range, aggregate rainfall, atmospheric pressure, relative humidity, wind velocity and sunshine hours, and SOI over 14 weeks. The Y lab represents the value of relative risk, the X lab represents the value of relevant variables. The red lines represent mean relative risks and the gray regions represent 95% CIs. The black vertical line represents the medians of the climatic factors and the SOI, and the dotted lines represent the 2.5 percentile and the 97.5 percentile for the climatic factors and the SOI, respectively.

The Figure 3 illustrated unidimensional summaries of the overall effects of meteorological factors and SOI on scrub typhus with the corresponding lag weeks. Non-linear relationships were observed. Briefly, for mean temperature, the RR and 95% CI values showed a rising trend, which were all over 1 from 23.5°C to 29.6°C. For WTR, the RR and 95% CI values revealed a decline trend, and it were over 1 from 8.1°C to 9.3°C and was <1 beyond 9.3°C. For aggregate rainfall, it was statistically significant from 0 to 70 mm, and the RR and 95% CI values were over 1 from 19 to 76 mm, with a peak at 38 mm. The RR values of atmospheric pressure were downtrend, with a peak at 989 hPa. For wind velocity, the RR values were uptrend and peaked at 1.6 m/s, then it went downtrend. For relative humidity, the RR values were uptrend and statistically significant from 45 to 72%. For SOI, the RR values showed an M-shape bimodal distribution, with a peak at 15.4. The statistically significant increase of RR value was observed in two stages from −16.2 to −4.4, and from 2.4 to 21.6. The RR and 95CI values were lower than 1 beyond 31.0. Finally, sunshine hours did not have a statistical correlation with scrub.

**Figure 4:**
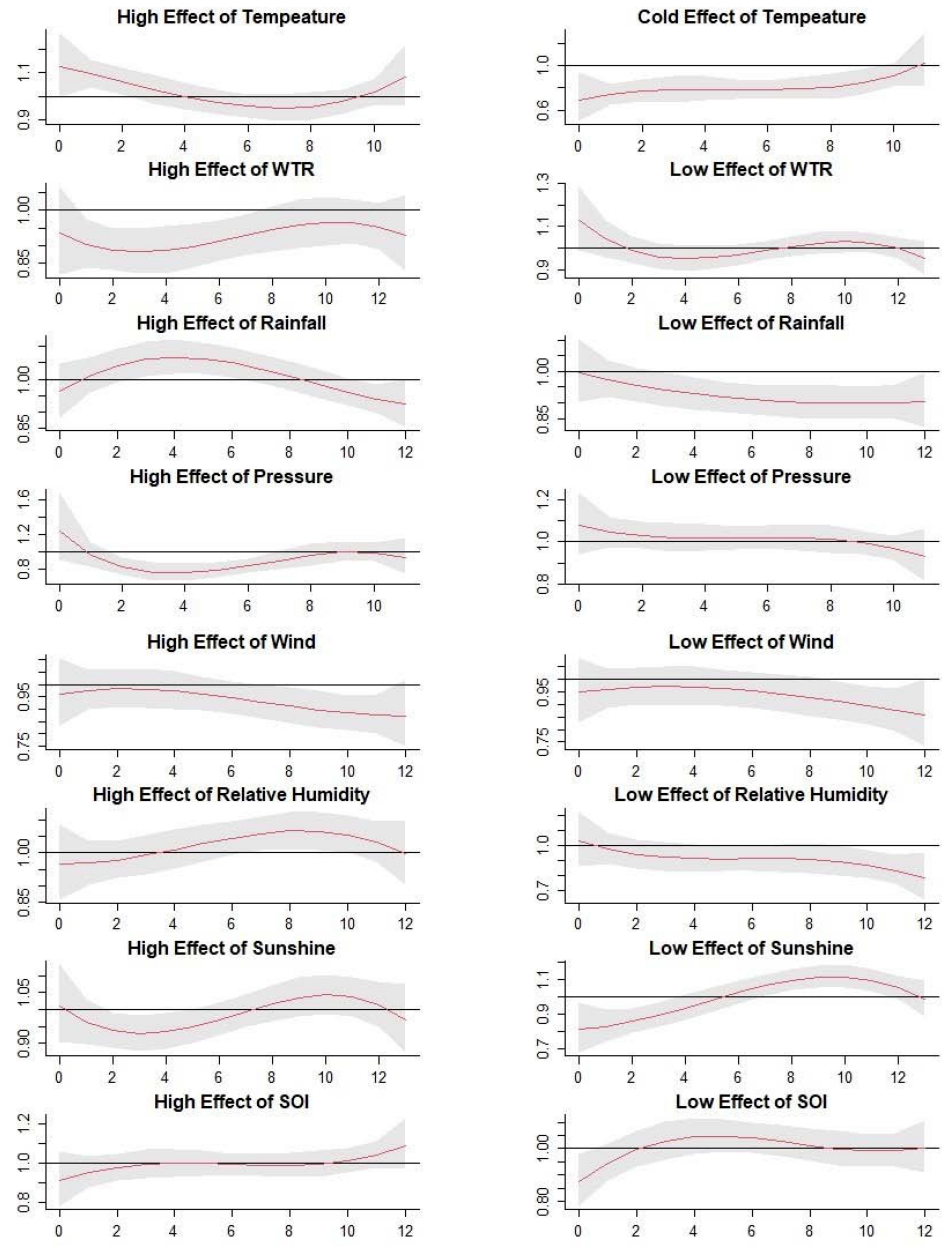
The estimated extreme effects of mean temperature, weekly temperature range, aggregate rainfall, atmospheric pressure, wind velocity, relative humidity, hours of sunshine, and SOI with extremely high effects (97.5%) and extremely low effects (2.5%). The Y lab represents the value of relative risk, the X lab represents the value of lag weeks. The red lines represent mean relative risks and gray regions represent 95% CIs.

The Figure 4 illustrated the extreme effects of meteorological factors and SOI on scrub typhus cases along the various lag weeks (Figure 4 and Table 2).

**Table 2:** The extreme effects of meteorological variables and SOI on scrub typhus along the lag weeks in Guangzhou, Southern China, 2006–2018.

**Meteorological variables**	**Lag week**	**High effect (97.5%)**		**Lag week**	**Low effect (2.5%)**	
		**RR**	**95%CI**		**RR**	**95%CI**
Mean temperature	1	1.10	1.04-1.16	0	0.69	0.50-0.94
Weekly temperature range	3	0.88	0.82-0.95	/	/	/
Aggregate rainfall	12	0.88	0.79-0.98	10	0.90	0.85-0.95
Atmospheric pressure	4	0.76	0.66-0.86	/	/	/
Wind velocity	11	0.88	0.80-0.96	12	0.86	0.73-0.99
Relatively humidity	8	1.07	1.01-1.12	12	0.78	0.64-0.95
Sunshine hours	3	0.93	0.88-0.98	0	0.81	0.67-0.97
Sunshine hours	/	/	/	10	1.12	1.05-1.19
SOI	/	/	/	0	0.88	0.78-0.98

*The median value of each variable was set as reference*.

The sensitivity analyses revealed that similar results were generated by changing the df for seasonality and long-term trends and meteorological factors in our study.

## Discussion

During the study period, we found that scrub typhus cases in Guangzhou showed a sharply rising trend, consistent with previous studies in China. Scrub typhus incidence increased stably before 2012, and then after 2012, it increased rapidly year by year (4, 7). However, the distribution of scrub typhus cases in Guangzhou was different, where the peak of cases occurred in 2012 and fluctuated at a stable level, although the numbers of cases increased again in 2018. Scrub typhus was a local notifiable disease by law in Guangzhou, this was a possible reason for the stability of occurrence. It demonstrated that the surveillance is beneficial to prevent and control the scrub typhus.

The high-risk occupations of scrub typhus in Guangzhou were farmer, house worker, and retiree. In Guangzhou, the retiree and house worker people would often visit the park and part of them would sit on the grass. The plausible explanation was that agricultural activities and park visiting would facilitate the exposure to pathogen-carrying chigger mites (19, 22). However, our speculation should be explored by the detailed epidemiology investigation to the scrub typhus cases in the future.

In our study, we found a seasonal fluctuation of scrub typhus cases from May to October which was not unexpected when compared to previous studies in the southern China (4, 7). However, it was not consistent with the study in Japan which was biphasic with the main peak in November and December and a smaller peak in May and June. This discrepancy was mainly caused by the different geography and climate.

Meteorological variables, including temperature, rainfall, and relative humidity have been demonstrated to get an important influence with time lags effect on the emergence and transmission of certain rodent-borne infectious diseases (12, 23). Lately, people paid more attention to the correlations between meteorological factors, SOI and scrub typhus, and DLNMs, which are widely used to explore the non-linear and lagged relationship, for example, a DLNM result in Taiwan revealed the non-linear and lag relationship between dengue fever and temperature (24). The time lags on scrub typhus may be caused by the life cycle of chigger mites (~2-3 months) (25) and the disease incubation period (average 10 to 12 days) (10).

In our study, we used weekly data to apply DLNMs to explore the relationships between meteorological factors, SOI and scrub typhus cases, which was more accurate comparing to previous studies by monthly data (19, 26). What's more, we added the autoregression term to reflect the infectious term in the DLNMs.

Our findings depicted that mean temperature was mainly positively correlated with scrub typhus cases before 29.6°C, which was consistent with previous studies in India (27) and Korea (26). Biological and ecological evidence can be found to support our findings. Temperature variation can influence the Trombiculidae spawning rates, the abundance and distribution of rodents and the activity of the chiggers. When the temperature increased, the Trombiculidae spawning rates and the abundance of rodents became higher and it promoted the activity of the chiggers (28). Furthermore, warmer condition was more adapted to farm and visit the park (19). Meanwhile, our findings showed that RR was highest when the temperature was 27°C with 2-week lag. While the temperature became hotter, people would reduce the willingness to farm or go outside such as a park. The reasons mentioned above supported our results, and the negative impact of atmospheric pressure on scrub typhus was approximately opposite to the effect of mean temperature, which was consistent with the result of a previous study using the negative binomial regression in Guangzhou (29). Unfortunately, the study about the impact of atmospheric pressure on scrub typhus was sparse, a possible reason for this phenomenon is that high atmospheric pressure is not conducive to the survival of mites (30), the biological mechanism should be undertaken in further study.

Temperature range was reported by previous studies to be related to infectious diseases, including influenza (31), hand foot mouth disease (32), and scrub typhus, although the study just pointed out that temperature range was a key determining factor (7). Our study revealed a negative relationship between the WTR and scrub typhus cases. The explanation was as follows. The high WTR usually occur in the winter season in Guangzhou, the Trombiculidae spawning rates and the abundance of rodents were lower and it weakened the activity of the chiggers and reduced the infectious risk of scrub typhus (28). The biological study should be launched in the future.

Previous studies showed that precipitation had a positive relationship with the occurrence of scrub typhus (10, 19, 26), which was partially in compliance with our finding. We found that the relative risk increased until the accumulate rainfall reached 40 mm, after which point, the relative risk started to decline. The positive effect on scrub typhus could be related to the increase in precipitation which could increase the growth of vegetation, which directly or indirectly facilitated the survival and reproduction of rodents, leading to higher rodent density (33). As the rainfall increased continuously over 70 mm, it would prohibit people to farm or go outdoor activity, which decreased the contact rate with the chiggers. The above reasons could explain our findings.

Relative humidity was shown to be positively correlated with the incidence of scrub typhus (10, 26). Our finding approximately matches with their results, showing a positive correlation with scrub typhus. However, as the relative humidity exceeded the 72%, the relationship was not significant. The biological reasons are as below. When the humidity is low, the adult chiggers stop their spawning. As the relative humidity increased, it offers a moist environment for the growth of mites (19, 29). A study in Chile shows that the number or activity of chigger mites decreased when the relative humidity is lower than 50%, as the relative humidity exceeds 50%, chigger mites can survive and reproduce well (34). However, as the continuous increase of relative humidity over 72%, it was often coincided with rainy day, and the effect of relative humidity may be offset by the effect of precipitation mentioned above. Hence, no statistically significant effect was observed after 72% relative humidity. This reason could partially explain our findings.

A study in Korea showed that the wind speed was correlated to scrub typhus (26). Our result illustrated that the RR rose sharply as the wind speed increased, however, after the wind speed exceeded 1.6 m/s, the RR dropper consecutively. The extremely high effect of wind speed showed the RR as 0.88. There was little research to explain the potential mechanism about the effect of wind velocity on scrub typhus. The study in Korea indicated the wind speed may be correlated to the chigger spawning condition (26). The gale weather is often accompanied by rain, which may influence the activity of people to farm or sit on the grass. However, further studies about the effect of wind speed on the ecology of the chigger lift cycle and infection mechanism should be done in future.

The Sunshine hours have been found in previous studies to have a positive association with scrub typhus cases (29, 35), however our result showed no statistical significance on the overall effect but extreme effect between the sunshine hours and scrub typhus. The RRs of extreme low effects of sunshine hours were 0.81 with 0-week lag and 1.12 with 10-week lag. When the duration of sunshine hours is short, it often coincided with precipitation, which reduces the exposure of people to the chiggers. The 2-week lag was consistent with the incubation of scrub typhus. Meanwhile, the precipitation was beneficial for the rodents and chigger to thrive (29). Hence, the RR increased and peaked in lag week 10. It gave us a hint that we should prepare for the control and prevention of scrub typhus when the storm season came.

The Southern Oscillation Index (SOI) reflects global features rather than local meteorological factors (14), which was useful for understanding the long-term trends of tsutsugamushi disease. When we took the larger geographic area climate factors into consideration, it could avoid the collinearity and make the trends more reasonable (36). Previous studies found that the SOI was correlated with some infectious diseases, including dengue fever, and chikungunya (37, 38). Our findings reported that the SOI showed an M-shaped curve relationship with scrub typhus cases. Sustained negative values of the SOI below −7 often indicate El Niño episodes, which would cause warmer and more precipitation in Guangzhou. While the sustained positive values over 7 are typical of a La Niña episode, which would cause cooler in autumn and winter in Guangzhou (39). The El Niño and La Niña episodes could cause the variation of temperature and precipitation (40). This finding was consistent with the distribution of Scrub typhus incidence in Guangzhou, Southern China, which was seasonal high from May to October and peaked in June. It was also in accord with our result about the mean temperature and aggregate rainfall, which would affect the density and activity of rodents and chiggers, also influence human behavior. For example, as the positive SOI value increased, the temperature would become cooler and cooler, and our result revealed that the RR values of SOI were below 1 beyond 31.0, and the RR values of mean temperature were lower than 1 below 23.5°C. However, the internal mechanism of the SOI to either meteorological variable in our study or scrub typhus incidence was complex (41), which need to be further explored as many other factors, including vector, social and human behavior factors, can influence the incidence of scrub typhus.

Some limitations should be mentioned. First of all, we just used the data in Guangzhou, Southern China, which cannot represent other regions. Secondly, the detailed epidemiology investigation about tract event and route of infection for each scrub typhus cases were not available, which affect our interpretation of the effect in the high-risk occupation people. Finally, potential confounding variables could not be excluded in our analyses, for example, host susceptibility, vector factors, population density, and geographic factors. Investigations for these limitations should be launched in further study.

## Conclusions

In general, meteorological factors and SOI played an important role in scrub typhus occurrence in Guangzhou. Non-linear relationships were observed in almost all the variables in our study. Approximately, mean temperature and relative humidity positively correlated to the incidence of scrub typhus, on the contrary to the atmospheric pressure, and the WTR. Aggregate rainfall and wind velocity showed an inverse-U relationship curve, whereas the SOI appeared the bimodal distribution. These findings provide preliminary, but basic information to better understand the epidemic trends of scrub typhus in Guangzhou, southern China. And the result facilitates the development of the early warning system by meteorological factors and SOI surveillance to strengthen the prevention and control of scrub typhus.

## Data Availability Statement

The raw data supporting the conclusions of this article will be made available by the authors, without undue reservation.

## Ethics Statement

This study got the permission from the ethics committee of the Guangzhou Center for Disease Control and Prevention. Our study did not involve any private and personal information about scrub typhus cases. The data was anonymous.

## Author Contributions

ZY: conceptualization and writing—review and editing. JL, YL, XM, and ML: data curation. JL and XM: methodology. ZY and ML: supervision. JL and ZY: validation. JL and YL: visualization. JL, YL, and XM: writing—original-draft. All authors contributed to the article and approved the submitted version.

## Conflict of Interest

The authors declare that the research was conducted in the absence of any commercial or financial relationships that could be construed as a potential conflict of interest.

## Publisher's Note

All claims expressed in this article are solely those of the authors and do not necessarily represent those of their affiliated organizations, or those of the publisher, the editors and the reviewers. Any product that may be evaluated in this article, or claim that may be made by its manufacturer, is not guaranteed or endorsed by the publisher.

## Abbreviations

SOI: Southern Oscillation Index
RR: relative risk
CI: confidence interval
WTR: weekly temperature range
DLNMs: distributed lag nonlinear models.
