# Corrigendum: Impact of Meteorological Factors and Southern Oscillation Index on Scrub Typhus Incidence in Guangzhou, Southern China, 2006–2018

**DOI:** 10.3389/fmed.2021.783395

**Published:** 2021-10-26

**Authors:** Jianyun Lu, Yanhui Liu, Xiaowei Ma, Meixia Li, Zhicong Yang

**Affiliations:** ^1^Department of Infectious Disease Control and Prevention, Guangzhou Center for Disease Control and Prevention, Guangzhou, China; ^2^Department of Public Health Emergency Preparedness and Response, Guangzhou Center for Disease Control and Prevention, Guangzhou, China; ^3^Guangzhou Center for Disease Control and Prevention, Guangzhou, China

**Keywords:** distributed lag non-linear models, meteorological factors, southern oscillation index, scrub typhus, early warning

In the original article, there was an error. A correction has been made to **Discussion**, paragraph 9. Several Chinese words were typed in bymistake and should have been deleted. The sentence should be: “When the humidity is low, the adult chiggers stop their spawning”

The authors apologize for this error and state that this does not change the scientific conclusions of the article in any way. The original article has been updated.

## Publisher's Note

All claims expressed in this article are solely those of the authors and do not necessarily represent those of their affiliated organizations, or those of the publisher, the editors and the reviewers. Any product that may be evaluated in this article, or claim that may be made by its manufacturer, is not guaranteed or endorsed by the publisher.

