# Supplementary material for: Impact of Meteorological Factors and Southern Oscillation Index on Scrub Typhus Incidence in Guangzhou, Southern China, 2006–2018
Source: Front Med (Lausanne). 2021 Jul 28;8:667549. doi: 10.3389/fmed.2021.667549 (PMC8355740; doi:10.3389/fmed.2021.667549)
Supplement: Supplementary file 1 [file Table_1.DOCX]

**Supplementary Table 1**

The detailed of the model structure for each variable in our study.

| Object variable | Other variables put in the model | AIC | df |
| --- | --- | --- | --- |
| TM | WTR+SOI | 3675.66 | 3 |
| WTR | TM+RH+Rain+SOI | 3901.23 | 3 |
| Rain | TM+WTR+SOI | 3868.67 | 4 |
| Pressure | TM+WTR+SOI | 3826.65 | 4 |
| RH | TM+SUN+SOI+WTR | 3884.92 | 3 |
| Wind | TM+WTR+RH+Rain+SOI | 3904.07 | 4 |
| SOI | TM+Rain+SUN+WTR+RH | 3930.79 | 4 |
| Sunshine | TM+SOI+RH+WTR | 3382.80 | 3 |

TM represents mean temperature; WTR represents weekly temperature range; Rain represents aggregate rainfall; Pressure represents atmospheric pressure; RH represents relative humidity; Wind represents wind velocity; SOI represents southern oscillation index; Sunshine represents duration of sunshine hours.
